# Sex differences influence intestinal epithelial stem cell proliferation independent of obesity

**DOI:** 10.14814/phy2.13746

**Published:** 2018-06-27

**Authors:** Weinan Zhou, Elizabeth A. Davis, Kailiang Li, Romana A. Nowak, Megan J. Dailey

**Affiliations:** ^1^ Department of Animal Sciences University of Illinois at Urbana‐Champaign Urbana Illinois; ^2^ Neuroscience Program University of Illinois at Urbana‐Champaign Urbana Illinois

**Keywords:** GI tract, obesity, proliferation, sex difference, stem cell

## Abstract

The intestinal epithelium is continuously regenerated by cell renewal of intestinal epithelial stem cells (IESCs) located in the intestinal crypts. Obesity affects this process and results in changes in the size and cellular make‐up of the tissue, but it remains unknown if there are sex differences in obesity‐induced alterations in IESC proliferation and differentiation. We fed male and female mice a 60% high‐fat diet (HFD) or a 10% low‐fat diet (LFD) for 3 months and investigated the differences in (1) the expression of markers of different intestinal epithelial cell types in vivo, and (2) lasting effects on IESC growth in vitro. We found that the growth of IESCs in vitro were enhanced in females compared with males. HFD induced similar in vivo changes and in vitro early growth of IESCs in males and females. The IESCs isolated and grown in vitro from females, though, showed an enhanced growth that was independent of obesity. To determine whether this effect was driven by sex steroid hormones, we used primary intestinal crypts isolated from male and female mice and investigated the differences in (1) the expression of steroid hormone receptors, and (2) cell proliferation in response to steroid hormones. We found that estrogen receptor *α* was expressed in crypts from both sexes, but estrogen had no effect on proliferation in either sex. These results suggest that obesity similarly effects IESCs in males and females, but IESCs in females have greater proliferation ability than males, but this is not driven by a direct effect of sex steroid hormones on IESCs or other crypt cells that provide essential niche support for IESCs.

## Introduction

The intestinal epithelium plays important roles in nutrient absorption, satiety hormone release, and immune barrier function. The epithelium is regenerated every few days in order to maintain the proper function of the tissue, a process that is driven by continuous cell renewal of intestinal epithelial stem cells (IESCs) localized near the base of intestinal crypts (Leblond and Stevens 1948; Barker et al. 2010). IESCs give rise to progenitor cells which, in turn, differentiate into mature intestinal epithelial cell types (e.g. Paneth cells, enterocytes, enteroendocrine cells, goblet cells). Obesity influences this process and results in changes in the size and cellular make‐up of the tissue (Dailey 2014). In particular, high‐fat diet‐induced obesity increases IESC number and proliferation, total epithelial cell number, crypt depth, or villus height in vivo (Baldassano et al. 2013; Mao et al. 2013; Mah et al. 2014; Beyaz et al. 2016) and drives lasting effects on IESCs, such that isolated IESCs from obese versus lean mice grow at different rates in vitro (Mah et al. 2014; Beyaz et al. 2016). These findings suggest that obesity may affect the growth and function of the intestinal epithelial tissue through modulating IESCs. However, most studies regarding the effect of obesity on IESCs and intestinal epithelial regeneration are skewed toward the use of male animals, without delineating the sex differences in obesity‐induced alterations.

Stem cells in many tissues are regulated in a sex‐specific manner. In mammals, sex‐specific regulation of stem cells has been found in hematopoietic (Nakada et al. 2014), neural (Pawluski et al. 2009), and muscle (Deasy et al. 2007) stem cells, such that stem cells in females exhibit increased stem cell number, proliferation, or regeneration potential compared with males. In *Drosophila*, IESCs in females exhibit greater proliferation, regenerative capacity, and organ size than in males (Hudry et al. 2016). That said, unique tissue and species‐specific stem cell properties are demonstrated and may not equate to similar sex‐induced changes in mammalian IESCs. Thus, we tested whether there were sex differences in the proliferation and differentiation of IESCs in mammals, and if obesity‐driven changes in IESCs differed between males and females. In Experiment 1, we fed male and female mice a 60% high‐fat diet (HFD) or a 10% low‐fat diet (LFD) for 3 months to investigate sex differences in obesity‐induced alterations in IESC proliferation and differentiation. We then measured changes in (1) crypt depth, villus height, and the expression of markers of different intestinal epithelial cell types in vivo, (2) lasting effects on IESC growth in vitro, and (3) proliferation of organoids that developed from IESCs in response to nutrient (i.e., glucose) application in vitro. In Experiment 2, we tested whether sex differences in IESC proliferation may result from the differential expression of steroid hormone receptors or steroid hormone‐induced proliferation because these mechanisms have been previously found to influence stem cell proliferation between the sexes (Pawluski et al. 2009; Nakada et al. 2014). We used primary small intestinal epithelial crypts, which include IESCs, progenitor cells and Paneth cells, isolated from male and female mice and investigated the differences in (1) the expression of estrogen receptor *α* (ER*α*), estrogen receptor *β* (ER*β*), progesterone receptor (PR), and androgen receptor (AR), and (2) proliferation in response to steroid hormone application in vitro.

## Materials and Methods

### Experiment 1

#### Animals

Male (*n* = 24) and female (*n* = 20) Lgr5‐EGFP‐ires‐CreERT2 mice at 10‐ to 12‐week‐old were used. These mice were bred at the Division of Animal Resources facility at the University of Illinois at Urbana‐Champaign from original breeding pairs obtained from the Jackson Laboratory (Bar Harbor, ME). Mice were individually housed in modified shoebox cages with a raised woven wire platform and lined with brown kraft paper for collection and measurement of food spillage. Mice were acclimated to the housing for 1 week with ad libitum access to tap water and laboratory chow (Teklad 22/5, Teklad Diets, Madison, WI) on a 12:12 light:dark cycle in a climate‐controlled room (22 ± 1°C and 60% relative humidity). All procedures were approved by the Institutional Animal Care and Use Committee at the University of Illinois at Urbana‐Champaign.

Following 1 week acclimation, mice were fed a HFD or LFD for 3 months. Male and female mice were divided into groups based on average body weight as follows: (1) 60% HFD fed (Research Diets D12492; *n* = 13 males; *n* = 11 females), and (2) 10% LFD fed (Research Diets D12450J; *n* = 11 males; *n* = 9 females). We have previously found that HFD‐induced obesity, and not just the HFD, is necessary to induce changes in IESC proliferation compared with lean, LFD mice (Zhou et al. 2017). Thus, we did not include a HFD fed group that was weight‐matched to the LFD group. Mice were weighed weekly and food intake was measured daily by weighing the food left in the hopper and subtracting the food spillage.

After 3 months, mice were killed by decapitation under isoflurane anesthesia (Henry Schein Animal Health, Dublin, OH). The mice were killed in the middle of the light cycle following a 6 h food deprivation. A subset of the mice (*n* = 3 per group) were used for isolation and culture of IESCs and another subset of mice (the rest of the mice in each group *n* = 6–10) were used for histology or qPCR. A power analysis (G*Power 3.1.9.2) based on previously published data investigating the lasting effect of diet‐induced obesity on IESC growth in vitro and epithelial changes in vivo show that to achieve a power = 0.8 and a type I error of 0.05, an *n* = 3 per group for in vitro experiments and an *n* = 5 per group for in vivo analysis are needed (Beyaz et al. 2016; Lee et al. 2018). The intestine was exposed and the entire small intestine was harvested for immediate IESC isolation. Another subset of mice (the rest of mice in each group *n* = 6–10) were used for histology or qPCR. For these mice, we exposed the intestine and collected the mid‐small intestinal tissue (i.e. jejunum) as previously described (Blackmore et al. 2017). Briefly, to ensure that we were collecting similar segments of the intestine for analysis between animals, we measured from the pyloric sphincter and ileocecal valve to isolate the middle of jejunum and collected two 5 mm segments to be processed for histology or qPCR. For histology, each of the 5 mm intestinal segments were fixed in 10% neutral‐buffered formalin for 24 h at room temperature (RT) and then stored in 70% ethanol at RT prior to paraffin embedding. For qPCR, each of the 5 mm intestinal segments were stored in RNAlater stabilization solution (Invitrogen, Carlsbad, CA) at −80°C prior to RNA extraction.

#### Isolation of small intestinal crypts

Small intestinal crypts were isolated as previously described (Zhou et al. 2018). Briefly, the entire small intestine was harvested, opened longitudinally and washed with cold 1× PBS to remove luminal contents. The villi were scraped off with a coverslip. The tissue was cut into 2–4 mm pieces with scissors and washed 5–10 times with cold 1× PBS until the supernatant was almost clear. Tissue fragments were incubated with 2 mmol/L EDTA (Fisher Scientific, Pittsburgh, PA) and gently rocked at 4°C for 30 min. After removal of EDTA, tissue fragments were washed with 1× PBS 3 times. The supernatant was then collected and passed through a 70‐*μ*m cell strainer (Corning, Corning, NY) and centrifuged at 300*g* at 4°C for 5 min. The cell pellet was resuspended with basal culture medium (Advanced DMEM/F‐12 Medium [Gibco, Grand Island, NY] containing 2 mmol/L GlutaMax [Gibco, Grand Island, NY], 10 mmol/L HEPES [Gibco, Grand Island, NY] and 100 U/mL Penicillin‐Streptomycin [Gibco, Grand Island, NY]) and centrifuged at 200*g* for 2 min to remove single cells. The isolated crypts remaining were used for immediate IESC isolation.

#### IESC isolation and culture

IESCs were isolated and cultured as previously described (Sato and Clevers 2013; Wang et al. 2013). Briefly, freshly isolated crypts from Lgr5‐EGFP‐ires‐CreERT2 mice were resuspended with single cell dissociation medium (basal culture medium containing 1× N2 [Gibco, Grand Island, NY], 1× B27 [Gibco, Grand Island, NY] and 10 *μ*M Y‐27632 [Sigma‐Aldrich, St. Louis, MO]) at 37°C for 45 min. During incubation the cell suspension was pipetted every 10 min. Dissociated cells were passed through a 40‐*μ*m cell strainer (pluriSelect, Leipzig, Germany), followed by a 20‐*μ*m cell strainer (pluriSelect, Leipzig, Germany) and centrifuged at 300 g at 4 °C for 5 min. Single, live IESCs were sorted as GFP^high^ by fluorescence‐activated cell sorting (FACS) with a BD FACS ARIA II sorter into single cell dissociation medium. Dead cells were excluded from the FACS with the viability dye propidium iodide (Invitrogen, Carlsbad, CA). Freshly isolated IESCs from each animal were embedded in Matrigel (Corning, Corning, NY) including 1 *μ*mol/L Jagged‐1 peptide (Ana Spec, Fremont, CA) at 500 IESCs/10 *μ*L, seeded on 96‐well plates (replicates of 5 wells per animal) and incubated in IESC culture medium (basal culture medium containing 1× N2, 1× B27, 1 mmol/L N‐Acetyl‐l‐cysteine, 50 ng/mL EGF [Gibco, Grand Island, NY], 100 ng/mL Noggin [PeproTech, Rocky Hill, NJ] and 500 ng/mL R‐Spondin‐1 [PeproTech, Rocky Hill, NJ]). Medium was changed every other day. Representative images were captured on day 3, 5, 7, 9, and 12 postplating using an Axio Vert.A1 inverted microscope (Zeiss, Oberkochen, Germany) fitted with an Axiocam 503 mono camera (Zeiss, Oberkochen, Germany). By day 3, isolated IESCs have proliferated to produce more IESCs and progenitor crypt cells (i.e., transit amplifying cells). By day 5, we begin to see the differentiated daughter cells that make‐up the villus epithelium (e.g., enterocytes and enteroendocrine cells). By day 7, budding from the spheroid begins and the organoid with its structural villi and crypts begins to be formed. Organoid size and number were quantified through analysis of captured images at 10× or 2.5× magnification, respectively. Organoid size was quantified using ImageJ software (NIH, Bethesda, MD). Organoid number was counted in each well.

#### Cell proliferation measurement

We have previously found that nutrient availability enhances intestinal organoid/crypt cell proliferation in vitro (Zhou et al. 2018). Thus, to determine if obesity impacts the ability of cells to respond to changes in nutrient availability, and if these obesity‐driven alterations differ between males and females, we investigated the proliferation of organoids that developed from IESCs in response to glucose. Isolated IESCs were cultured for 12 days to form organoids (replicates of 5 wells per group per animal). Organoid cultures were changed to glucose‐free IESC culture medium (SILAC™ Advanced DMEM/F‐12 Flex Medium [Gibco, Grand Island, NY] containing 147.5 mg/L l‐arginine [Sigma‐Aldrich, St. Louis, MO], 91.25 mg/L l‐Lysine [Sigma‐Aldrich, St. Louis, MO], 2 mmol/L GlutaMax, 10 mmol/L HEPES, 100 U/mL Penicillin‐Streptomycin, 1× N2, 1× B27, 1 mmol/L N‐Acetyl‐l‐cysteine, 50 ng/mL EGF, 100 ng/mL Noggin and 500 ng/mL R‐Spondin‐1), and incubated for 4 h (Cognard et al. 2013; Blackmore et al. 2017). Organoids were then incubated either in IESC culture medium without glucose or with low (5.5 mmol/L) or high (17.5 mmol/L) concentrations of glucose (Sigma‐Aldrich, St. Louis, MO) with 5% CO_2_ at 37°C for 1 day. These concentrations were chosen based on hepatic portal vein or systemic glucose levels between and after meals in mice (Zhou et al. 2018). Cell proliferation was then measured using Cell Proliferation Reagent WST‐1 (Roche Diagnostics, Indianapolis IN) according to the manufacturer's instructions.

#### Histology

Morphometric analyses were performed on formalin fixed, paraffin embedded, and hematoxylin stained cross sections. Briefly, intestinal sections (5 mm) were fixed in 10% neutral buffered formalin for 24 h at RT and then stored in 70% ethanol at RT until later processing. In order to paraffin embed the tissue, sections were dehydrated in a series of graded ethanols, cleared in xylene and infiltrated with paraffin in a Tissue Tek VIP 1000 tissue processor (Miles, Elkhart, IN). Paraffin blocks of tissue were cut on a microtome at 5 *μ*m thickness and mounted onto charged glass microscope slides. Tissue sections were then deparaffinized in xylenes, hydrated in a series of graded ethanols, and rinsed in deionized water. Sections were stained in hematoxylin (Fisher HealthCare, Houston, TX) followed by dehydration in a series of graded ethanols and cleared in xylene. Slides were coverslipped with Permount mounting media (Fisher Scientific, Fair Lawn, NJ). Intestinal tissue sections were visualized using a NanoZoomer Digital Pathology (NDP) System (Hamamatsu, Hamamatsu City, Japan) using a 40× objective and NDP Scan software. Nine crypts or villi from the jejunal sections per mouse were chosen based on intact morphology of the intestine (Argenzio et al. 1990; Heise et al. 1991; Liu et al. 2015; Jinga et al. 2017). Crypt depth and villus height were measured using NDP View 2 software.

#### qPCR

To determine the expression of markers of different intestinal epithelial cell types in tissue, total RNA was extracted from tissue using RNeasy Plus Mini Kit (Qiagen, Hilden, Germany). RNA was reverse transcribed into cDNA using QuantiTect Reverse Transcription Kit (Qiagen, Hilden, Germany). qPCR was performed with TaqMan Universal PCR Master Mix (Applied Biosystems, Foster City, CA) and TaqMan probes (Applied Biosystems, Foster City, CA) using Applied Biosystems QuantStudio™ 7 Flex Real‐Time PCR System. Negative reverse‐transcribed samples were generated and all reactions were carried out in triplicate. The following TaqMan probes were used: alkaline phosphatase, intestinal: Mm01285814_g1, chromogranin A: Mm00514341_m1, Lgr5: Mm00438890_m1, lysozyme 1: Mm00657323_m1, mucin 2: Mm01276696_m1 and GAPDH: Mm99999915_g1. To determine relative expression values, the 2^−ΔΔCt^ method was used, where triplicate Ct values for each sample were averaged and subtracted from those derived from GAPDH.

### Experiment 2

#### Animals

Male (*n* = 3) and female (*n* = 3) C57BL/6J mice at 10‐week‐old were used. These mice were obtained from the Jackson Laboratory (Bar Harbor, ME), and were maintained with ad libitum access to tap water and laboratory chow (Teklad 22/5, Teklad Diets, Madison, WI) on a 12:12 light:dark cycle in a climate‐controlled room (22 ± 1°C and 60% relative humidity). All procedures were approved by the Institutional Animal Care and Use Committee at the University of Illinois at Urbana‐Champaign. Male and female mice were killed by decapitation under isoflurance anesthesia. Female mice were killed at the diestrus stage of the estrous cycle (when estradiol levels are low) to minimize the variability in hormone levels during the estrous cycle (Patel et al. 2017). For both male and female mice, the chest cavity was opened and blood was collected from the right ventricle of the heart for immediate serum separation, which was then stored at −20°C prior to serum estradiol level measurement. The intestine was then exposed and the entire small intestine was harvested for immediate crypt isolation.

#### Isolation of small intestinal crypts

Small intestinal crypts were isolated as described above. The isolated crypts were used for qPCR or cell proliferation measurement. Day 1 of isolated crypts includes the IESCs and progenitor cells. By day 3 of crypt culture, organoids with villus and crypts were present.

#### qPCR

To determine the expression of steroid hormone receptors in isolated crypts, total RNA was extracted from isolated crypts using RNeasy Plus Mini Kit (Qiagen, Hilden, Germany). To determine the expression of ER*α* in response to 17*β*‐estradiol in crypts, total RNA was extracted from crypts embedded in Matrigel using RNeasy Plus Universal Mini Kit (Qiagen, Hilden, Germany). Reverse transcription, qPCR, and determination of relative expression values were performed as described above. The following TaqMan probes were used: ER*α*: Mm00433149_m1, ER*β*: Mm00599821_m1, AR: Mm00442688_m1, PR: Mm00435628_m1 and GAPDH: Mm99999915_g1.

#### Cell proliferation measurement

For determination of the proliferation in response to 17*β*‐estradiol, freshly isolated crypts from each animal were embedded in Matrigel at 200 crypts/10 *μ*L, seeded on 96‐well plate (replicates of 4 wells per group per animal), incubated in phenol red‐free IESC culture medium (glucose‐free IESC culture medium containing 17.5 mmol/L glucose). Crypts were then incubated either in phenol red‐free IESC culture medium with 0.1% ethanol (control) or with different concentrations of 17*β*‐estradiol (1 or 10 nmol/L) with 5% CO_2_ at 37°C for 1 or 3 day. These concentrations were chosen based on effective physiological doses found to elicit changes in intestinal epithelium and in the proliferation of estrogen receptor positive cell types (Lippman et al. 1976; Brunner et al. 1989; Picotto et al. 1996; Chow et al. 2004; Strom et al. 2004; O'Mahony et al. 2007). MCF‐7 estrogen receptor positive cells (American Type Culture Collection, Rockville, MD) were used as the positive control. MCF‐7 cells were seeded at 10,000 cells/well in 96‐well plates (replicates of 4 wells per group), incubated in MCF‐7 culture medium (phenol red‐free MEM [Gibco, Grand Island, NY] containing 5% charcoal stripped fetal bovine serum [Gibco, Grand Island, NY], 0.1 mmol/L nonessential amino acids [Gibco, Grand Island, NY], 2 mmol/L GlutaMax, 1 mmol/L sodium pyruvate, 10 *μ*g/mL insulin and 100 U/mL Penicillin‐Streptomycin), and cultured for 1 day. MCF‐7 cells were then incubated either in MCF‐7 culture medium with 0.1% ethanol (control) or with different concentrations of 17*β*‐estradiol (1 or 10 nmol/L) with 5% CO_2_ at 37°C for additional 1 or 3 days (Lippman et al. 1976; Brunner et al. 1989; Chow et al. 2004). 17*β*‐estradiol or 0.1% ethanol was added to crypts or MCF‐7 cells daily. Cell proliferation was then measured using Cell Proliferation Reagent WST‐1.

#### Serum estradiol levels

Estradiol levels in collected sera were measured using Mouse/Rat Estradiol ELISA kit (Calbiotech, El Cajon, CA) according to the manufacturer's instructions.

#### Data analysis

Data are expressed as Mean ± SEM. For measurements of body weight and food intake, differences between groups were analyzed using a two‐way RM ANOVA. For measurements of crypt depth, villus height, the expression of markers of different intestinal epithelial cell types, organoid size, organoid number, and ER*α* expression and cell proliferation in response to 17*β*‐estradiol, differences between groups at each time point were analyzed using a two‐way ANOVA. For measurement of cell proliferation in response to glucose, differences between groups were analyzed using a three‐way ANOVA. For measurement of MCF‐7 cell proliferation in response to 17*β*‐estradiol, differences between groups at each time point were analyzed using a one‐way ANOVA. Fisher's LSD post hoc tests were used where appropriate. For measurement of the expression of steroid hormone receptors and serum estradiol levels, differences between groups were analyzed using a Student's *t* test. *P* < 0.05 was considered statistically significant. Assumptions of normality, homogeneity of variance, and independence were met for all significant data.

## Results

### Body weight and food intake

**Figure 1:**
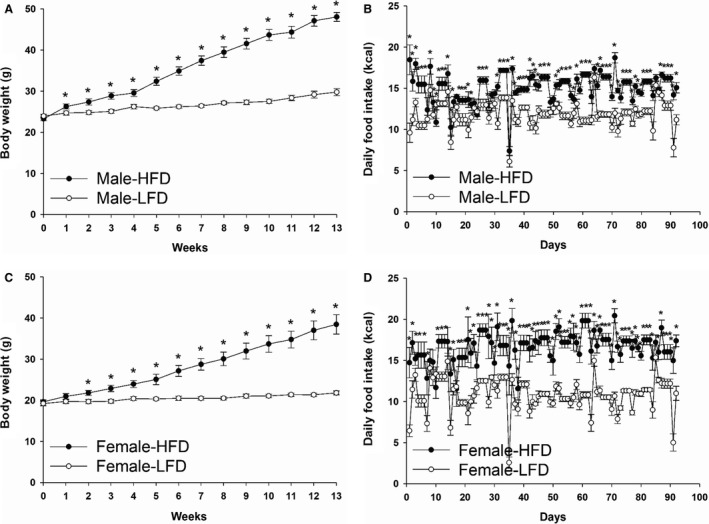
Body weight and daily food intake of male (A and B) or female mice (C and D) fed with HFD or LFD for 3 months. Data are expressed as Mean ± SEM (*n* = 9–13). * indicates significantly different at each time point, *P* < 0.05.

Body weight and food intake were increased in the HFD fed mice compared with the LFD group in male (Fig. 1A and B; *P* < 0.05) and female mice (Fig. 1C and D; *P* < 0.05).

### Intestinal crypt and villus morphometry

**Figure 2:**
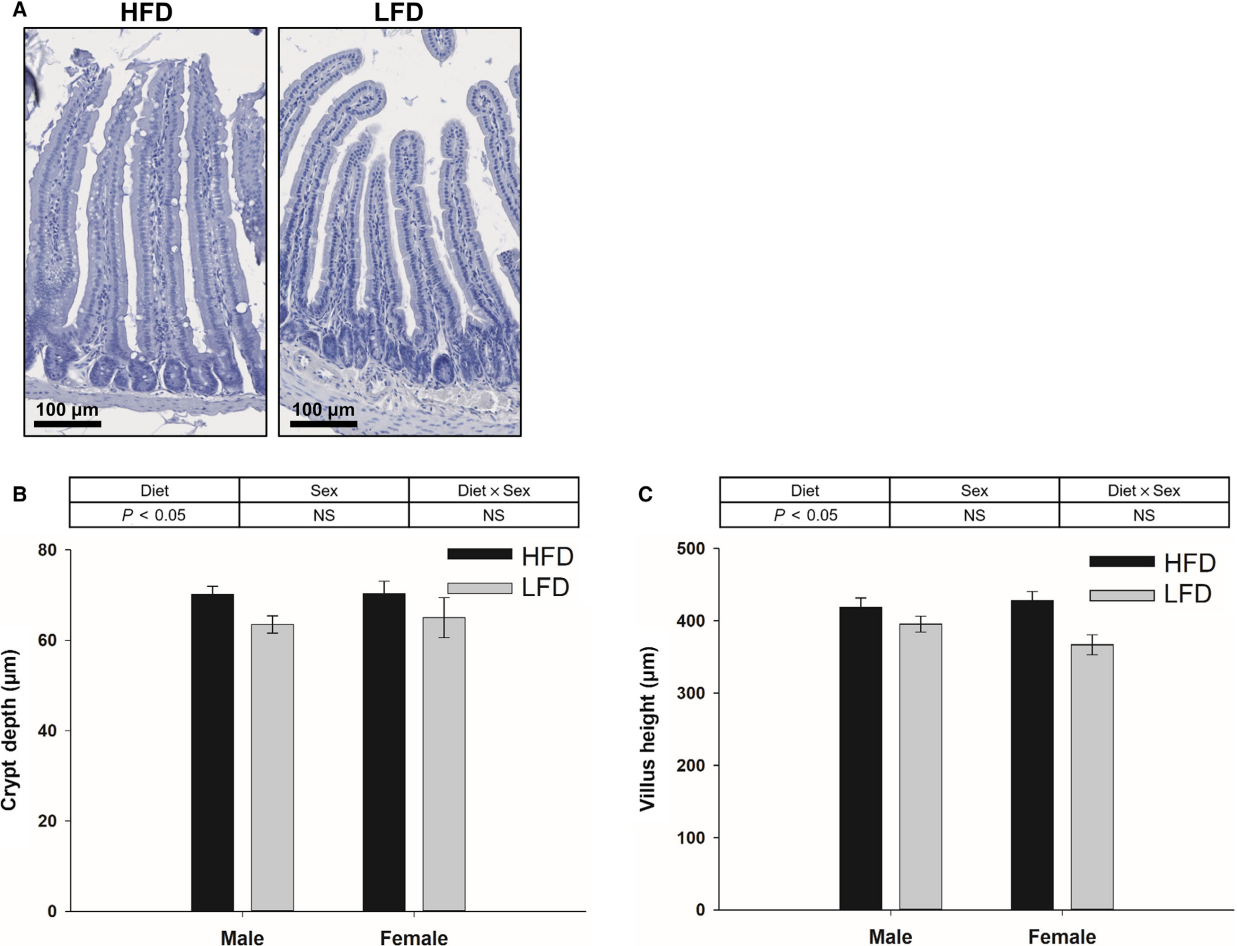
Representative image of hematoxylin stained crypt‐villus morphology (A) and quantification of crypt depth (B) and villus height (C) in the jejunum collected from mice fed with HFD or LFD using diet and sex as two factors (two‐way ANOVA). Data are expressed as Mean ± SEM (*n* = 6–10). Scale bar, 100 *μ*m.

Crypt depth and villus height were greater in HFD fed mice than LFD group (Fig. 2A–C; *P* < 0.05), but there was no sex effect or interaction between diet and sex (Fig. 2B and C).

### Expression of intestinal epithelial cell markers

**Figure 3:**
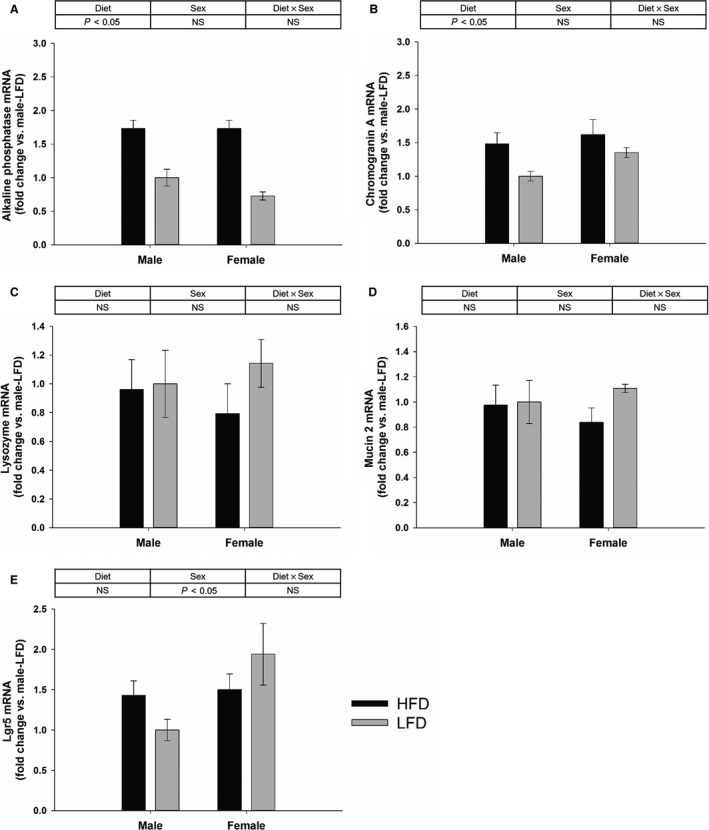
Gene expression of markers of different intestinal epithelial cell types in the villi and crypts of the jejunum collected from mice fed with HFD or LFD using diet and sex as two factors (two‐way ANOVA). Gene expression of alkaline phosphatase (enterocytes) (A), chromogranin A (enteroendocrine cells) (B), lysozyme (Paneth cells) (C), mucin 2 (goblet cells) (D), and Lgr5 (IESCs) (E). Data are expressed as Mean ± SEM of fold change relative to the male‐LFD group (*n* = 6–10).

The expression of alkaline phosphatase (enterocyte marker) and chromogranin A (enteroendocrine cell marker) were increased in HFD fed mice compared with LFD group (Fig. 3A and B; *P* < 0.05), but there was no sex effect or interaction between diet and sex (Fig. 3A and B). There was no diet effect, sex effect or interaction between diet and sex on the expression of lysozyme (Paneth cell marker) and mucin 2 (goblet cell marker) (Fig. 3C and D). The expression of Lgr5 (IESC marker) was greater in females than males (Fig. 3E; *P* < 0.05), but there was no diet effect or interaction between diet and sex (Fig. 3E).

### IESC growth and proliferation in response to glucose in vitro

**Figure 4:**
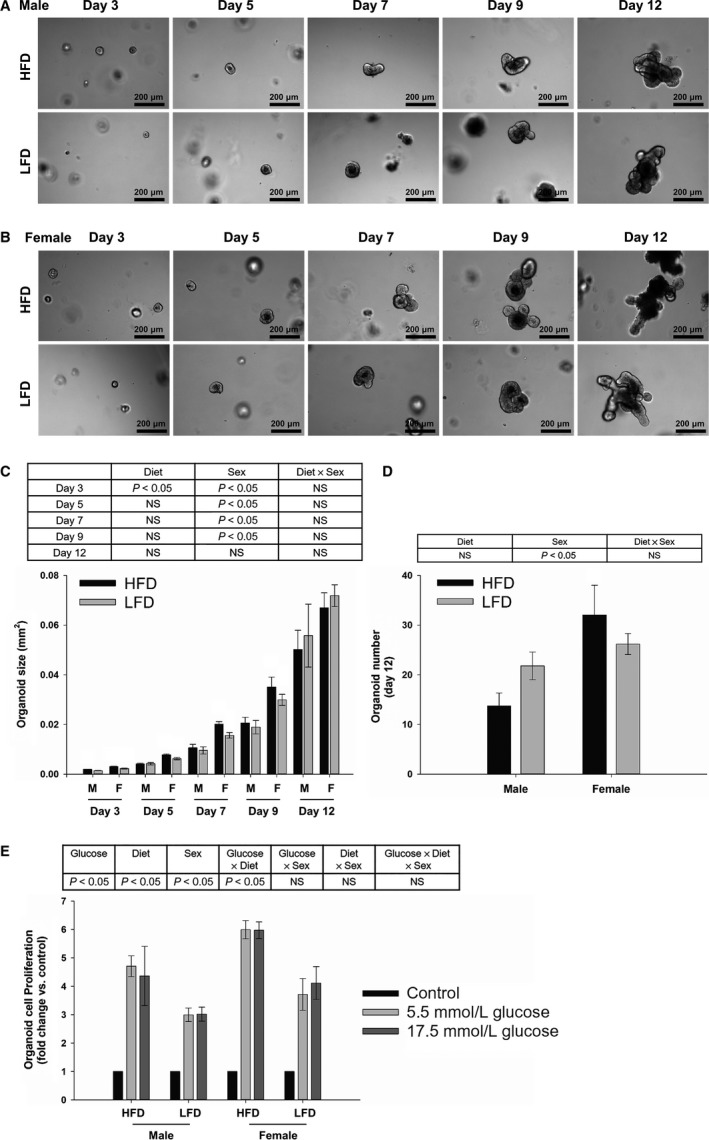
Representative images and quantification of the size and number of organoids that developed from IESCs isolated from male (A, C, and D) or female (B, C, and D) mice fed with HFD or LFD using diet and sex as two factors (two‐way ANOVA). Data are expressed as Mean ± SEM (*n* = 3). Scale bar, 200 *μ*m. Cell proliferation of day 12 organoids in response to glucose (E) using glucose, diet and sex as three factors (three‐way ANOVA). Data are expressed as Mean ± SEM of fold change relative to the control group (*n* = 3).

The size of the organoids that developed from IESCs isolated from HFD fed mice was greater than those isolated from the LFD group on day 3 postplating (Fig. 4C; *P* < 0.05). The size of the organoids that developed from isolated IESCs was greater in females than males on days 3, 5, 7, and 9 postplating (Fig. 4C; *P* < 0.05). There was no interaction between diet and sex on any day (Fig. 4C). The number of organoids that developed from IESCs on day 12 post plating was greater in females than males (Fig. 4D; *P* < 0.05), but there was no diet effect or interaction between diet and sex (Fig. 4D). When these day 12 organoids were then exposed to varying levels of glucose, proliferation was greater in the 5.5 and 17.5 mmol/L glucose conditions compared with the control group, was greater in HFD group than LFD group, and was greater in females than males (Fig. 4E; *P* < 0.05). There was an interaction between glucose and diet (Fig. 4E; *P* < 0.05), but there was no interaction between glucose and sex, between diet and sex, or between glucose, diet and sex on the proliferation (Fig. 4E).

### Expression of steroid hormone receptors in isolated crypts and serum estradiol levels

**Figure 5:**
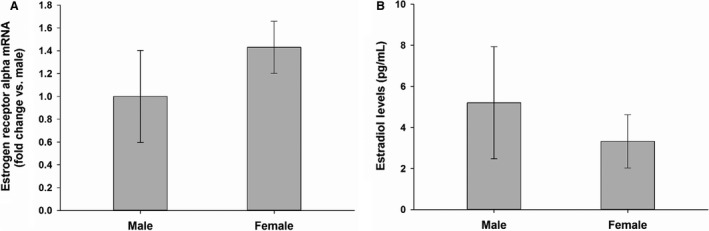
The expression of steroid hormone receptors in isolated intestinal crypts (A) and serum estradiol levels (B). Data are expressed as Mean ± SEM of fold change relative to the male group (*n* = 3) for qPCR, data are expressed as Mean ± SEM (*n* = 3) for serum estradiol measurement.

ER*α* was expressed in isolated crypts from both male and female mice without sex differences (Fig. 5A), but ER*β*, AR, and PR were not expressed in either sex (data not shown). There were no differences in the serum estradiol levels between male and female mice at sacrifice (Fig. 5B).

### Cell proliferation in response to estrogen

**Figure 6:**
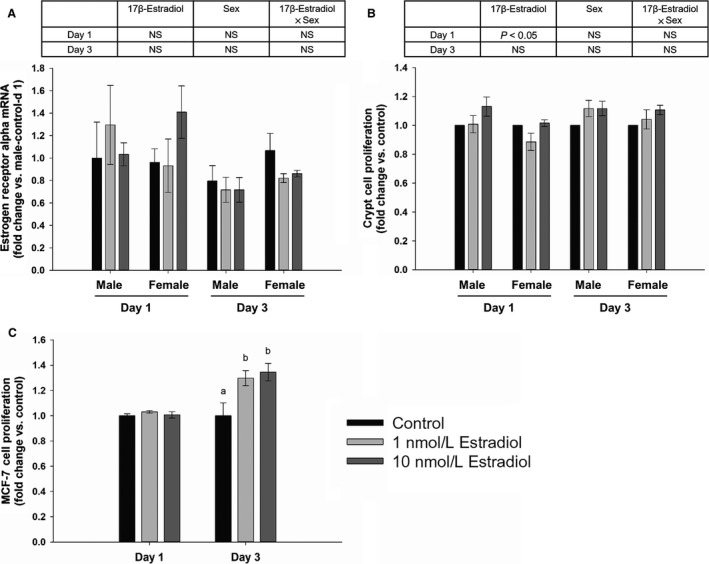
ER *α* expression (A) and cell proliferation (B) in response to 17*β*‐estradiol using 17*β*‐estradiol and sex as two factors (two‐way ANOVA;* n* = 3), and the proliferation of MCF‐7 cells (positive control) (one‐way ANOVA;* n* = 4) (C). Data are expressed as Mean ± SEM of fold changes. Means with different letters indicate significantly different at each time point, *P* < 0.05.

ER*α* was expressed in crypts in both male and female mice after 1 or 3 day 17*β*‐estradiol application, but there was no 17*β*‐estradiol effect, sex effect or interaction between 17*β*‐estradiol and sex on the expression of ER*α* (Fig. 6A). Cell proliferation was greater in 10 nmol/L 17*β*‐estradiol condition than 1 nmol/L 17*β*‐estradiol condition after 1 day application (Fig. 6B; *P* < 0.05), but 1 or 10 nmol/L 17*β*‐estradiol condition was not different from the control group (Fig. 6B). There was no sex effect or interaction between 17*β*‐estradiol and sex on crypt proliferation after 1 day 17*β*‐estradiol application (Fig. 6B). There was no 17*β*‐estradiol effect, sex effect or interaction between 17*β*‐estradiol and sex on crypt proliferation after 3 day 17*β*‐estradiol application (Fig. 6B). 1 and 10 nmol/L 17*β*‐estradiol increased the proliferation of MCF‐7 estrogen receptor positive cells after 3 day application (Fig. 6C; *P* < 0.05).

## Discussion

The aim of these experiments was to investigate whether there are sex differences in the proliferation and differentiation of IESCs in mammals, if obesity‐driven changes in IESCs differ between males and females, and whether steroid hormones contribute to these sex differences. The major findings were as follows: (1) HFD induced similar in vivo changes in females as has previously been found in males, (2) HFD enhanced the early growth of IESCs in vitro in females as has previously been found in males, (3) the growth of IESCs in vitro was enhanced in females compared with males, independent of HFD, (4) HFD and sex‐induced changes in proliferation remained when challenged with a new nutrient (i.e., glucose) environment, (5) ER*α*, but not ER*β*, PR, and AR, was expressed in both male and female crypts, and (6) estrogen had no effect on proliferation in either sex.

A myriad of differences in the morphology and function of the intestinal epithelium have been documented between obese and lean individuals, but whether these changes are similarly found in males and females has not explicitly been investigated previously. We have replicated findings that obese humans and nonhuman animal models have greater villus height and crypt depth and that this increase in epithelial mass is reflected by an increase in the number of enterocytes similarly in males and females (Mayer and Yannoni 1956; Kageyama et al. 2003; Verdam et al. 2011). Because enterocytes comprise the vast majority of the epithelial cells, any change in their numbers would likely result in significant functional differences, which has previously been seen as an obesity‐induced increase in absorptive capacity (Mayer and Yannoni 1956; Singh et al. 1972; Balint et al. 1980; Ferraris and Vinnakota 1995). In addition to the increase in enterocytes, we found that the increase in epithelial size may be driven by an increase in enteroendocrine cells. Whereas some studies find a similar obesity‐induced increase in enteroendocrine cells (Aranias et al. 2015; Widmayer et al. 2015; Dusaulcy et al. 2016), others find no change (Mah et al. 2014; Beyaz et al. 2016) or decreases in their numbers (Richards et al. 2016; Wolnerhanssen et al. 2017). Obesity‐induced changes in goblet and Paneth cell number between studies have also been found (Yang et al. 2003; Mah et al. 2014; Beyaz et al. 2016). When comparing only the HFD‐induced mouse studies (and not those that include obesity developed through the use of transgenic models or ventromedial hypothalamic lesions), differences between studies likely reflect the type of diet [60% HFD (Widmayer et al. 2015; Beyaz et al. 2016; Dusaulcy et al. 2016; Richards et al. 2016) or 45% HFD (Mah et al. 2014)], the comparator diet [low‐fat (Dusaulcy et al. 2016) or chow (Mah et al. 2014; Widmayer et al. 2015; Beyaz et al. 2016; Richards et al. 2016)], or the length of time on the diet [4 months (Dusaulcy et al. 2016; Richards et al. 2016), 6 months (Mah et al. 2014; Widmayer et al. 2015) or longer (Beyaz et al. 2016)]. Because the intestinal epithelial cells are the first point of contact with ingested food and prepare the body for the nutrients that are entering the system, it makes sense that their cellular make‐up would be comprised of cells that would be best suited for the digestion of a diet that is maintained across time. The high adaptability of the epithelium to change its cellular make‐up is likely due to a coordinated effort of the presence and nature of the distinct food components (e.g., proteins, lipids, carbohydrates) in the lumen of the intestinal tract mixed with signals from the basal surface of the tissue (e.g., hormonal, neural). These signals likely direct the progenitor cells at the crypt‐villus axis to differentiate into the functional cells needed. A role for Notch signaling in directing epithelial lineage allocation has been outlined (Jensen et al. 2000; Yang et al. 2001). Mouse atonal homolog 1 (Math1) and hairy and enhancer of split 1 (Hes1) are two downstream transcription factors from Notch signaling that work in opposition to each other to drive the differentiation into secretory cells (i.e., enteroendocrine and goblet cells) or enterocytes, respectively (Jensen et al. 2000; Yang et al. 2001). Downstream of Math1, neurogenin3 directs enteroendocrine differentiation from goblet cells (Jenny et al. 2002). Morbidly obese patients that have undergone gastric bypass surgery show changes in the transcription of these factors and resulting differentiated daughter cell composition 3 months after surgery that is similar to that of lean individuals, even though the postgastric bypass patients are still obese (Wolnerhanssen et al. 2017). This suggests that the combination of luminal dietary components and basal signals that may drive changes in these transcription factors and epithelial composition is independent of body weight – a finding that has previously been proposed (Altmann and Leblond 1970; Steinbach et al. 1994; Dailey 2014). It still has yet to be elucidated, though, how the molecular mechanisms control epithelial cell differentiation and how dietary signals may mediate changes in these processes.

The impact of obesity to induce lasting consequences on stem cell proliferation and/or differentiation has been previously found [e.g., IESCs (Mah et al. 2014; Beyaz et al. 2016), bone marrow‐derived mesenchymal stem cells (Wu et al. 2013), hematopoietic stem cells (Lee et al. 2018), and adipose‐derived stem cells (Wu et al. 2013; Baptista et al. 2015)]. The conditions necessary for acquisition of the functional and molecular changes in stem cells appear to be progressive in nature. That is, the more extreme the obesity due to the length of time on a HFD or the transgenic obese model used appears to result in a more extreme phenotype in the stem cells, a phenotype that can persist after weight loss, after transplantation into a normal environment, or after isolation and in vitro culture of the stem cells (Wu et al. 2013; Mah et al. 2014; Baptista et al. 2015; Beyaz et al. 2016; Lee et al. 2018). These previous findings in other types of stem cells follow with what we found in IESCs. Although we have replicated the findings of Beyaz et al. (Beyaz et al. 2016) that a 60% HFD‐induced obesity enhanced IESC growth in vitro compared with those isolated from lean mice, the length of time this effect lasted was different. We found that IESCs isolated from mice on a 60% HFD for 3 months retained in vitro differences in growth for only 3 days, whereas when mice were maintained on the diet for 9–14 months, the in vitro differences remained for much longer (Beyaz et al. 2016). These data suggest that the length of time an animal is obese or maintained on a HFD may drive changes in the IESC niche to alter proliferation and growth of the tissue. Mah et al. (Mah et al. 2014; Andres et al. 2015; Van Landeghem et al. 2015) postulate that the lasting consequences of obesity on IESCs are driven by obesity‐associated high circulating levels of insulin and local release of insulin‐like growth factor‐1 (IGF‐1). The changes in insulin/IGF‐1 signaling in IESCs may then promote progressive epigenetic modifications in the proliferation process that are determined by the degree of metabolic disturbances associated with the obesity. The mice used in the present experiment and the other 60% HFD‐induced obesity experiment (Beyaz et al. 2016), are a good model to mimic the human metabolic derangements observed in obesity. Hyperglycemia, hyperinsulinemia, and insulin resistance can be seen as early as 4 weeks on a HFD, but can become progressively worse as the length of time on the HFD is maintained for longer durations (Inui 2003; Sato et al. 2010; Wang and Liao 2012). The aberrant insulin receptor signaling that occurs as a result of obesity, including Akt and mTOR signaling, is known to alter cell proliferation and growth and may mediate progressive changes in the epigenetic control of genes related to the cell cycle. This model of how obesity may promote lasting consequences in proliferation has yet to be directly tested, though.

We show for the first time that sex differences impact IESC proliferation (independent of HFD‐induced obesity), which is shown as greater IESC growth in vitro. In particular, we found that there were no sex differences in the size of the crypt‐villus in vivo, but when we isolated and cultured IESCs in vitro, a sex effect on IESC growth became apparent and lasted for multiple days. It is likely that in vivo, the IESCs were probably in a similar niche environment (e.g., high insulin, IGF‐1) for a long period of time. Therefore, the cells from males and females have similarly adapted, which lead to no sex differences in tissue size in vivo. When we moved the cells from in vivo to in vitro, they were being moved into a new environment. Because we plated equal numbers of cells from each group, there must be some intrinsic differences in how the male versus female cells respond to the new environment, which lead to the sex differences in vitro. This initial difference between male and female cells in how they proliferate in vitro disappeared so that there were no significant differences on day 12. When we took a subset of these “similar” acting cells on day 12 and transferred them to yet another new environment, the sex difference reappeared. We found that male and female cells differentially responded to a new glucose environment. This sex difference, though, may be driven by a difference in the number of organoids between the sexes on day 12, which is something that we could not control. We cannot explain why there are more organoids in females, but the size of the organoids is the same in females and males. Taken together, there must be some intrinsic differences in how the male versus female cells adapt to a new environment.

Although sex steroid hormones have been found to directly contribute to stem cell proliferation of select tissues (Pawluski et al. 2009; Nakada et al. 2014), we did not find that to be the case for IESCs. We found that the IESCs and other crypt cells lacked ER*β*, PR and AR and only expressed ER*α* in both males and females. We also found no induction of proliferation with the application of estrogen even though the doses used induced proliferation in an estrogen sensitive cell line. This suggests that sex differences in IESCs do not result from short‐term, direct effects of steroid hormones on IESCs or other crypt cells which provide essential niche support for IESCs. This estrogen‐independent manner of action has been found in the regeneration of skeletal muscle stem cells (Deasy et al. 2007). Moreover, although sex steroids do not directly alter IESC proliferation, sex steroids may play an indirect role through modulating the stem cell niche as has been found in other stem cells (Calvi et al. 2003; Zhang et al. 2003; Qiu et al. 2014). Stromal cells underlying the intestinal epithelium within the lamina propria modulate the function of the IESCs (Kabiri et al. 2014; Tan and Barker 2014) and express estrogen receptor (Press et al. 1986). Thus, circulating estrogen may indirectly regulate stem cell proliferation and differentiation thorough modulating stromal cells of lamina propria in the stem cell niche.

These results suggest that there is a differential IESC proliferative capacity in males versus females in response to changes in their nutrient environment, but this difference is independent of HFD‐induced obesity and not driven by a direct effect of steroid hormones on IESCs or other crypt cells that provide essential niche support for IESCs. This finding is relevant to treatments used to remedy abnormal proliferation or function of the intestinal epithelium because the IESCs may differentially respond to acute changes in nutrient availability or pharmacological approaches aimed at altering proliferation and differentiation. A further understanding how the sex difference in cellular adaptation may affect tissue regeneration or function may help to create tools to positively and differentially manipulate the tissue between the sexes.

## Conflict of Interest

The authors declared no potential conflicts of interest.
